# The Virological and Immunological Characteristics of the HIV-1-Infected Population in Brazil: From Initial Diagnosis to Impact of Antiretroviral Use

**DOI:** 10.1371/journal.pone.0139677

**Published:** 2015-10-28

**Authors:** Ricardo Sobhie Diaz, Lilian A. Inocêncio, Maria Cecilia Araripe Sucupira, Anderson Alvarenga Pereira, James Hunter, João Eduardo Ferreira, Luciano V. Araújo, Denise F. C. Souza, Ester Cerdeira Sabino

**Affiliations:** 1 Federal University of São Paulo, São Paulo, Brazil; 2 Brazilian STD/AIDS and Viruses Hepatitis Department, Ministry of Health, Brasilia, DF, Brazil; 3 University of Sao Paulo, Sao Paulo, Brazil; Centro Nacional de Microbiología - Instituto de Salud Carlos III, SPAIN

## Abstract

**Background:**

Immunological and virological status of HIV-infected individuals entering the Brazilian public system over time was analyzed. We evaluated the impact of ART on virological, immunological and antiretroviral resistance over time.

**Methods:**

CD4+ T cell counts, viral loads and genotypes from patients over 13 years old from 2001–2011 were analyzed according to demographic data. We compared groups using parametric t-tests and linear regression analysis in the R statistical software language.

**Results:**

Mean baseline CD4+ T cell counts varied from 348 (2003) to 389 (2009) and was higher among women (p = 1.1 x 10^−8^), lower in older patients (p< 1 x 10^−8^) and lower in less developed regions (p = 1.864 x 10^−5^). Percentage of treated patients with undetectable viral loads increased linearly from 46% (2001) to 77% (2011), was lower among women (p = 2.851 x 10^−6^), younger ages (p = 1 x 10^−3^), and in less developed regions (p = 1.782 x 10^−4^). NRTI acquired resistance was 86% in 2001–3 and decreased over time. NNRTI resistance increased from 2001-3(50%) to 2006–9 (60%), PI resistance decreased from 2001–3 (60%) to 2009 (40%), and 3-class resistance was stable over time around 25%. Subtype prevalence comprised B (75.3%), B/F recombinants (12.2%), C (5.7%), F (5.3%) and B/C recombinants (1.5%), with regional variations. Three-class resistance was 26.5% among Bs, 22.4% among Fs and 17.2% among Cs.

**Conclusions:**

HIV diagnosis occurs late, especially among elderly Brazilians. Younger individuals need special attention due to poor virological response to treatment. Antiretroviral Resistance profile is subtype related.

## Introduction

Brazil has 757,042 reported AIDS cases as of December 2014. More than a decade ago, Brazil took a major step in the fight against HIV/AIDS by making antiretrovirals available free of cost to all infected citizens. As of October 2014, almost 400,000 individuals were under antiretroviral treatment (ART) out of almost 589,000 diagnosed HIV-infected individuals (http://www.aids.gov.br/publicacao/2014/boletim-epidemiologico-2014). As a result, AIDS-related mortality rates, which peaked in 1995/1996, have continually declined [1]. Given the sequential use of ART and the extensive use of unboosted protease inhibitors at the beginning of this program, we assume the proportion of patients experiencing virological failure to be high. One small Brazilian study showed the median time the viral load (VL) stayed below the detection limits during an initial treatment was approximately 14 months among treatment-naïve patients [2], while another study with a limited number of patients revealed that only 27.5% of the patients maintained undetectable VLs after one year of follow-up [3].

The public health system enables all HIV-infected individuals to receive monitoring and HIV testing, such as VL, CD4+ T cell determinations and HIV genotype testing upon virological failure. Previous studies have reported high levels of antiretroviral secondary resistance [4]. One major concern regarding viremic individuals with resistant viruses is the transmission of drug-resistant strains.

The Brazilian population presents several HIV subtypes, including clades B, F and C; a number of circulating recombinant forms, such as CRF_28B/F, CRF_29B/F, CRF_31B/C, CRF_38B/F, CRF_39B/F and CRF_46B/F (http://www.hiv.lanl.gov/content/sequence/HIV/CRFs/CRFs.html) as well as several unique recombinant forms, which may result from lower adherence among young adults due to more disordered lifestyles.

There is a growing epidemic of clade C and CRF_31B/F originating in the far south and moving north. However, clade B prevails in the Southeast region, the epicenter of the HIV epidemic in Brazil [5]. Half of the clade B Brazilian strains are genetically and antigenically distinct from typical clade B strains because they harbor the unique GWGR motif at the tip of the loop, which allegedly leads to lower cytopathogenicity [6]. The antiretroviral response and pathways of genotypic resistance are also of great interest in non-clade B strains, since clade B viruses cause only 10% of HIV infections in the world.

This study analyzed the baseline immunological and virological status of HIV-infected individuals entering the Brazilian public system over time. We also evaluated the impact of ART on viral suppression, immunological status and the antiretroviral resistance profile upon ART failure over time.

## Methods

We analyzed a central data bank from the STD/AIDS division of the Brazilian Ministry of Health containing 2,607,825 CD4+ T cell determinations and 2,483,055 viral loads (VLs) from patients over 13 years of age from 2001–2009 and treatment responses until 2011. Results from pregnant women have been excluded from this analysis for basal CD4+ T cell counts, since HIV testing among pregnant women is compulsory in Brazil, and the immunological status of pregnant women does not reflect the overall status of HIV-1 infected women.

The above-mentioned data bank receives information on CD4+ T cell counts, viral load and genotypes only from public centers, which contain almost all the data from HIV patients followed in Brazil. The database automatically captures the results of above tests from the laboratory network of the Brazilian Ministry of Health. Submission to the database of the data on antiretroviral treatment is mandatory for all Brazilian patients, including those followed in the private health sector. The data bank compiles this information for its internal use and it is not available to public. We gathered all epidemiological and raw data used in these analyses and deposited them at https://github.com/jameshunterbr/HIVProfileBrazil, with open access to public.

To determine basal CD4+ T cell counts and viral loads, we analyzed only the first results available for each patient who was not on ART. There is no data on the time of HIV-1 diagnosis and on the time elapsed from diagnosis to first CD4+ T cell count determination. However, local guidelines recommend that CD4 determinations should be performed as soon as HIV infection diagnosis is determined. To evaluate the virological and immunological antiretroviral response, we selected one entry (the first) for individuals with more than six months of treatment each year. Therefore, the number of samples analyzed corresponds to the number of patients analyzed. VL determinations in the Brazilian public system derive from a single commercial methodology according to national regulations. We considered VLs to be below detection limits if there were <400 RNA copies/mL of plasma. ART adherence Information for specific patients was not available.

We studied a total of 18,849 genotypes from distinct individuals experiencing virological antiretroviral failure between 2001 and 2009 [7]. Antiretroviral resistance related mutations from DNA sequences were determined using the tool developed for the Brazilian Algorithm for HIV resistance interpretation at http://forrest.ime.usp.br:3001/resistencia. Only major mutations were analyzed from the mutation list provided by the IAS-USA [8]. To avoid duplications, a blast search was performed at http://189.28.129.134/; if a pair of sequences with similarity above 99% was detected, only one of the duplicated sequences was included for analysis. HIV-1 subtype assignment was performed using the Los Alamos Recombinant Identification Program (RIP) (http://hivweb.lanl.gov/RIP/RIPsubmit.html). Analyzed sequences can be accessed at GenBank, accession numbers KT741029—KT748519.

Samples were considered to have resistance to 3 antiretroviral classes if they had at least one major mutation associated with resistance to nucleoside analogue reverse transcriptase inhibitors (NRTI), non-nucleoside analogue reverse transcriptase inhibitors (NNRTI), and protease inhibitors (PI) [9].

We analyzed the virological data according to gender, age and regions. Brazil contains 5 geographic regions: the more developed and wealthier Southeast region, followed by the South, Central-west, Northeast and North regions. Information on transmission HIV routes was not available for analysis.

We performed linear regressions on the data that indicated trends and t-tests to compare groups using the R statistical software system [10]. For all analyses, we considered the level of significance to be an alpha of 0.05. The study was approved by ethics committee at the Federal University of Sao Paulo/Brazil under the number 1433/09. As informed consent was not obtained, we anonymized and de-identified patient records/information prior to analysis.

The evidence shows that late presentation for care is prevalent in Brazil, since all available data was used. Therefore, the distribution of individuals by gender, age and country regions in the samples analyzed is compared with the corresponding information available in the Brazilian HIV/AIDS reports for the same time period (http://www.aids.gov.br/publicacao/2014/boletim-epidemiologico-2014).

## Results

**Fig 1:**
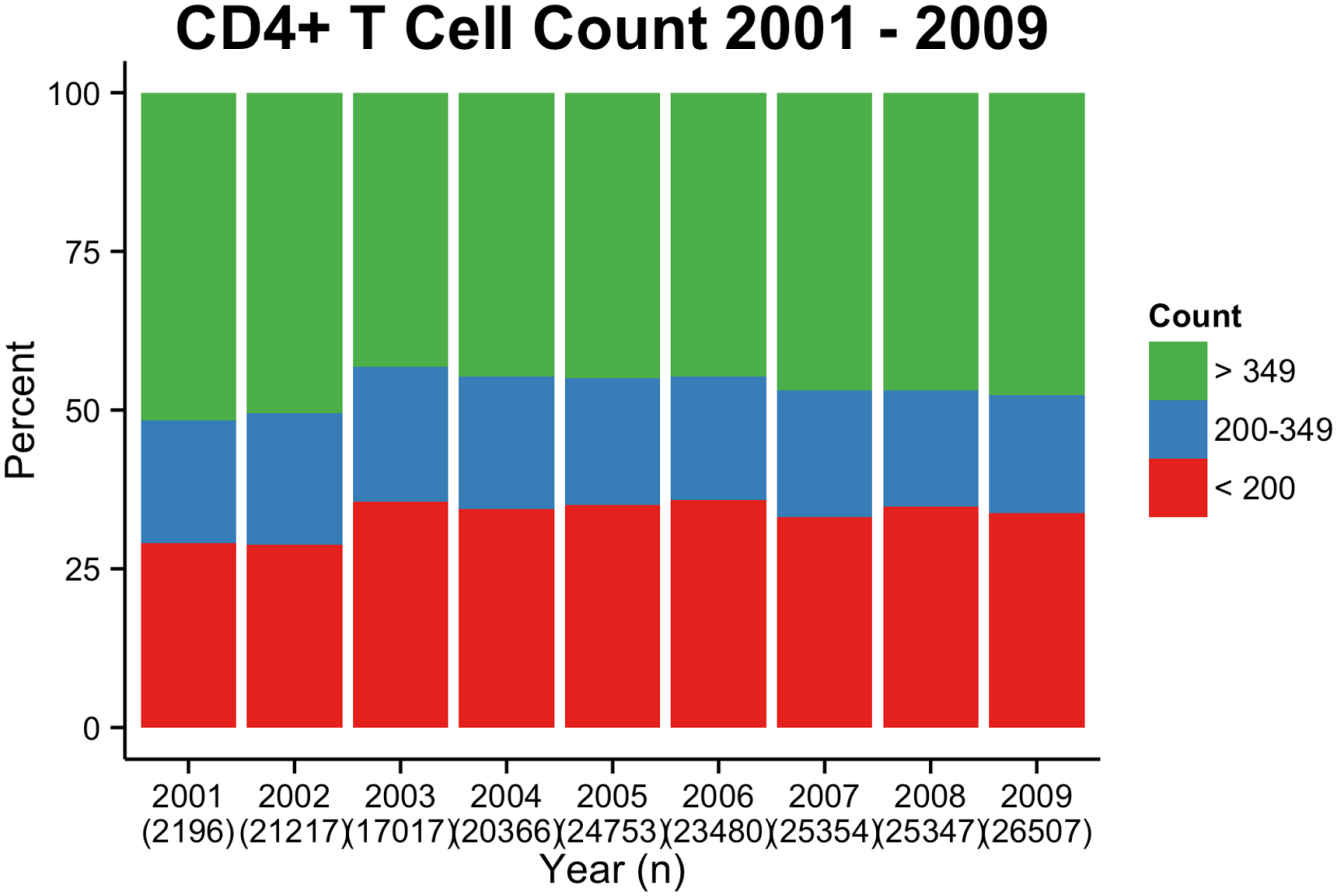
CD4 cell counts at initial screening. CD4+ T cell counts among antiretroviral naïve individuals at first presentation from 2001 to 2009.

We analyzed the immune status of patients who presented for care for the first time. Only the first CD4+ T cell count for each antiretroviral naïve individual from 2000 to 2009 was analyzed (n = 63,107), representing the year each patient started follow-up medical care. The mean CD4+ T cell counts were stable from 2001 to 2009, varying from 348 (2003) to 389 (2009) (Fig 1 and S1 Table). Half of patients presented with CD4+ T cell counts below 350/mm^3^ (48.36% in 2001 and 52.33% in 2009). One-third of patients presented with CD4+ T cell counts below 200/mm^3^ (29.01% in 2001 and 33.81% in 2009. Fig 1 and S1 Table).

**Fig 2:**
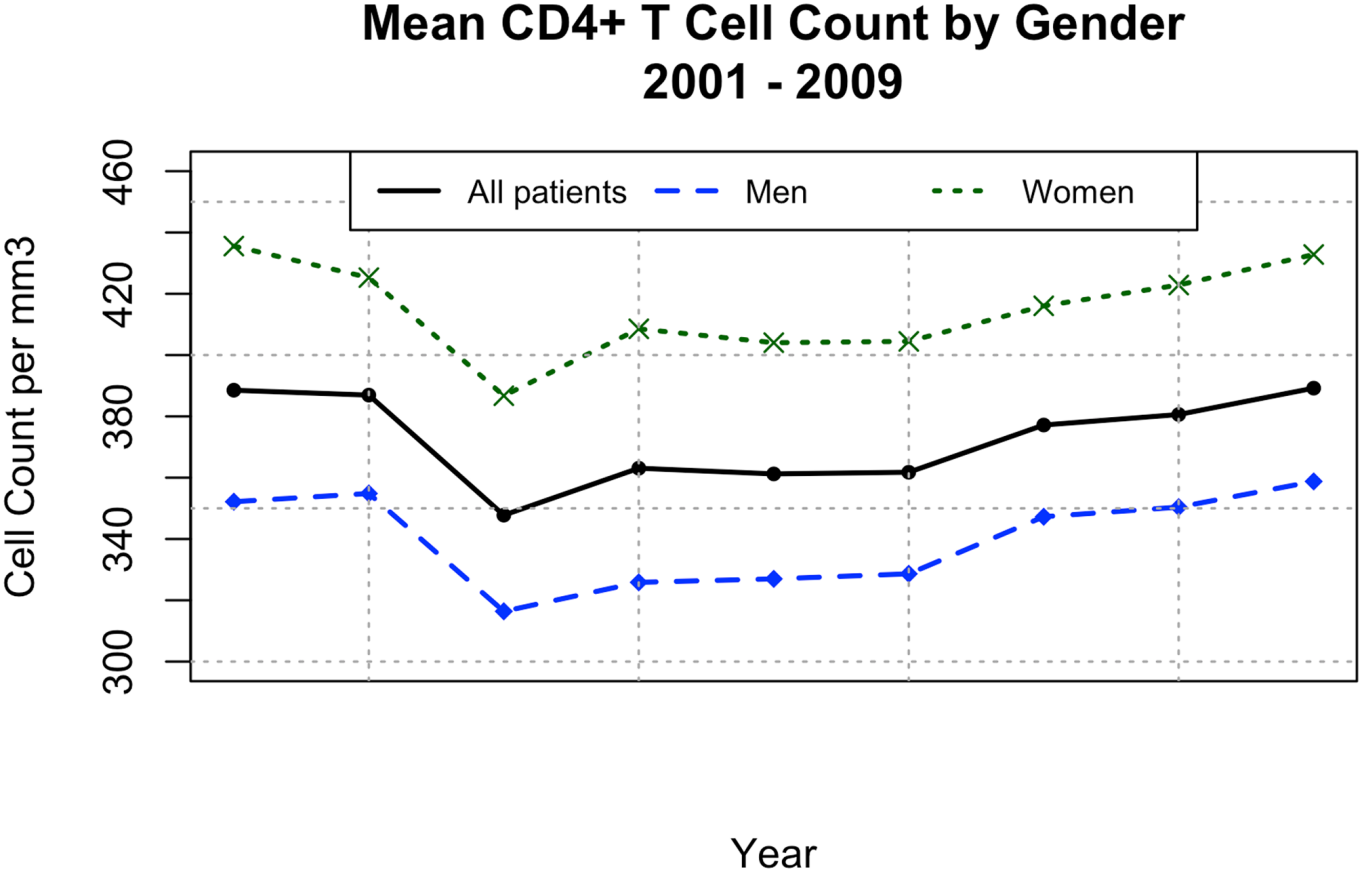
CD4 cell counts by gender. Mean CD4+ T cell counts by gender according to the first measurement for each patient from 2001 to 2009.

**Fig 3:**
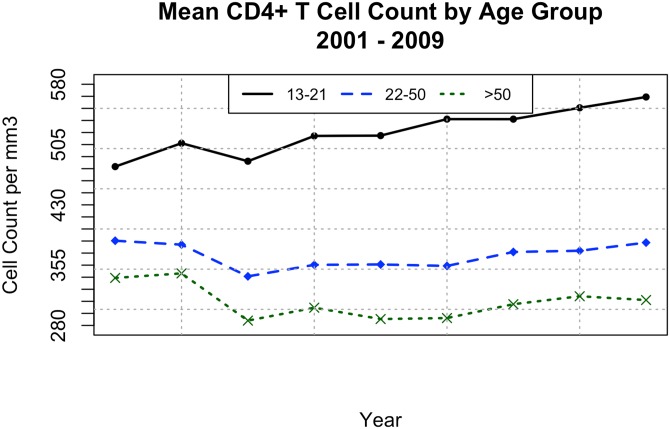
CD4 cell counts by age. Mean CD4+ T cell counts by age according to the first measurement for each patient from 2001 to 2009.

The test compared the scores of CD4+ T cell counts between genders and found that the men were consistently lower on a one-sided t-test (t = -10.1694 and a p-value of 1.1 x 10^−8^. Cf., Fig 2). CD4+ T cell counts also varied according to age (Fig 3). A one-sided t-test comparing ages 13–21 with ages 22–50 showed a significant difference—younger patients higher—with (t = 14.0573 and a p-value of < 1 x 10^−8^). Older patients (> 50 years old) presented with significantly lower CD4+ levels than the 22–50 group (t = 6.5047, p-value = 5.67 x 10^−6^). Half the individuals over 50 years old presented with CD4+ T cell counts below 200/mm^3^ (S2 Table).

**Fig 4:**
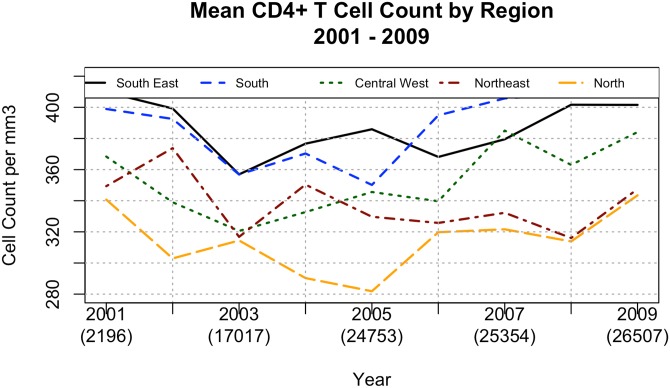
CD4 cell counts by region. Mean CD4+ T cell counts by region according to the first measurement for each patient from 2001 to 2009.

Immunological status varied according to the geographical regions, with higher CD4+ T cell counts in the Southeast and South regions, followed by the Central West, North and Northeast regions (Fig 4). We first conducted an analysis of variance to test the significance of the differences among regions. This analysis showed that region was a significant factor in CD4+ levels (F = 20.013, df of 4 and 40 and a p-value of < 1 x 10^−8^). We also conducted a t-test comparing the Southeast and the North regions (those with the greatest difference in means). This confirmed that CD4+ T cell counts between these regions is significantly different (t = 7.9771 with 15.662 df and a p-value = 1.864 x 10^−5^).

**Fig 5:**
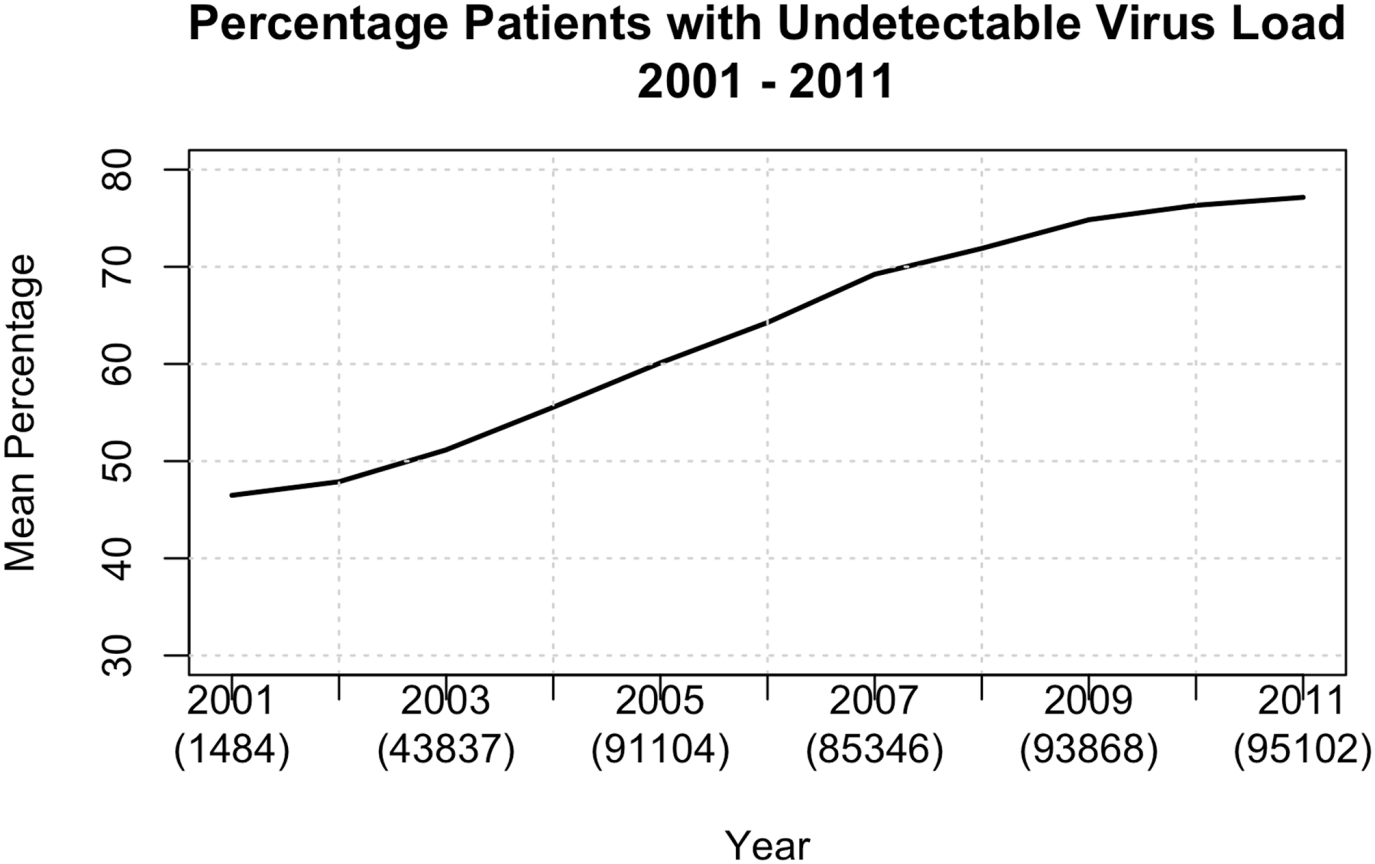
Viral loads below detection limit. Percentage of viral loads below detection among all individuals on antiretroviral treatment by age from 2001 to 2011.

**Fig 6:**
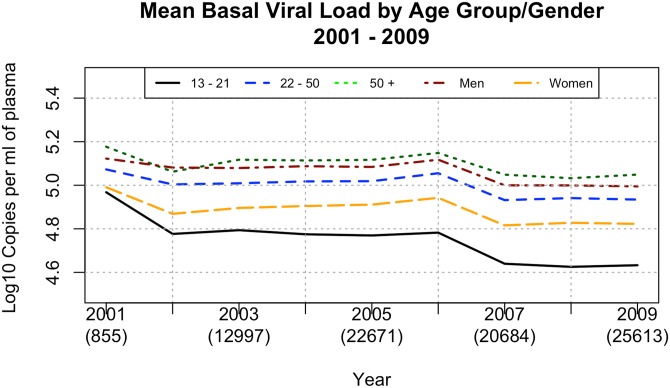
Mean basal viral load. Mean basal viral load by age and gender according to the first measurement for each patient from 2001 to 2009.

We assessed the viremia of antiretroviral-naïve individuals according to the first available RNA VL for 90,859 individuals, as shown in Fig 5. As distinct laboratories in the Brazilian Public Health System perform CD4+ T cell counts and viral loads, the number of patients analyzed for CD4+ T cell counts and VLs are different. Furthermore, VL results from pregnant women have been included in this analysis but not in the basal CD4+ T cell counts analysis. The basal VLs tended to fluctuate by approximately 5 log_10_ copies/mL of plasma, and also tended to be higher among men (t = 6.7633 with 15.326 df and p-value = 2.851 x 10^−6^), and lower among older patients (t = 4.029 with 15.348 df and p-value = 5 x 10^−4^) (Fig 6).

**Fig 7:**
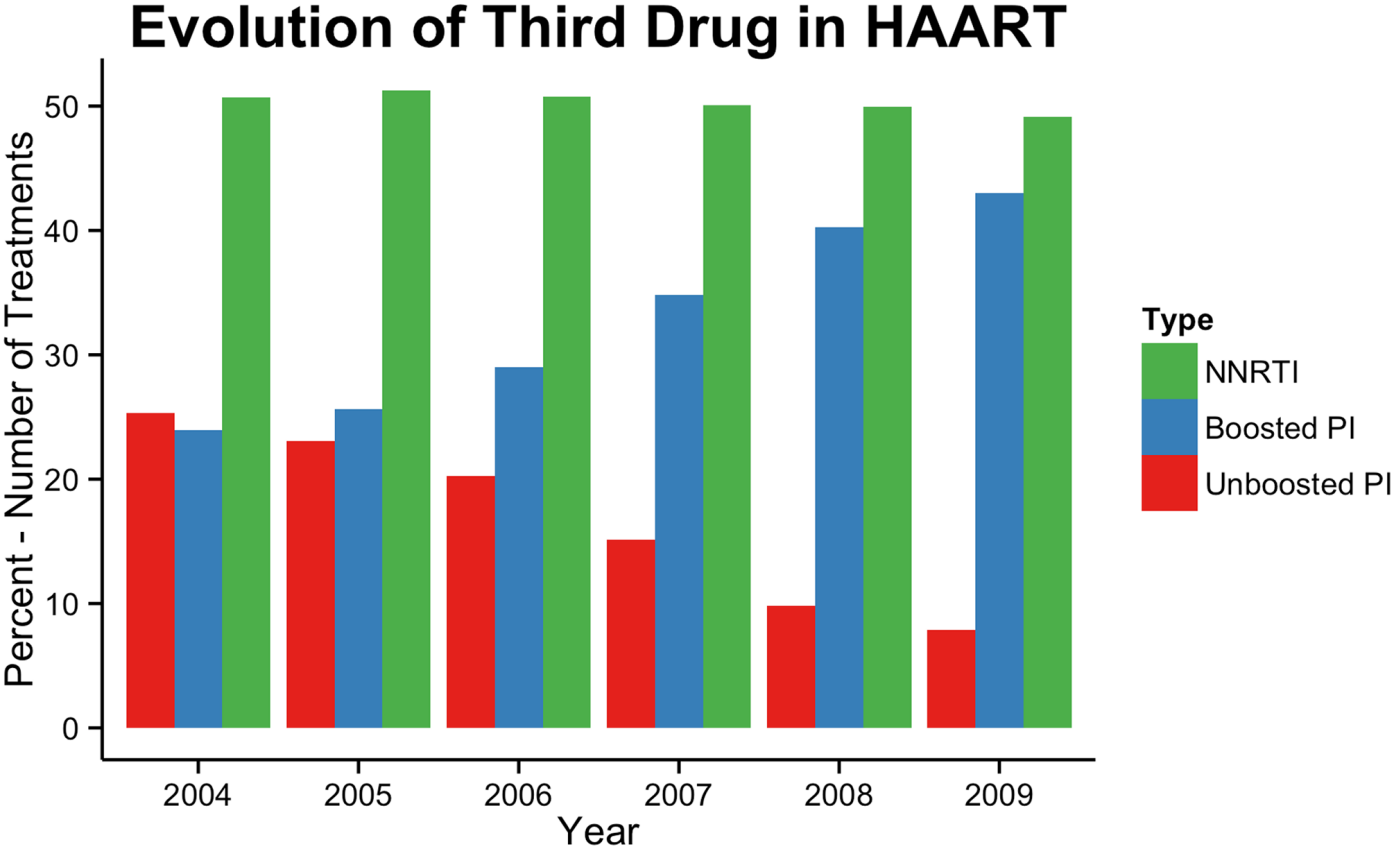
Evolution of third ART treatment over time. Number of treatments containing non-nucleoside reverse transcriptase inhibitors (NNRTIs), unboosted protease inhibitors (PIs) and ritonavir-boosted PI from 2004 to 2009.

We next evaluated the antiretroviral efficacy in treated individuals in Brazil. To do so, we evaluated the VL and CD4 results for individuals in treatment from 2001 to 2011. First, we were able to detect differences in antiretroviral exposure since the third drug used in highly active antiretroviral therapy (HAART) has dramatically changed over time, specifically when unboosted PIs gave way to NNRTIs and boosted PI-based regimens, as shown in Fig 7. We also detected a linear increase in the percentage of VLs below 400 copies of genomic RNA/mL of plasma, from 46.5% in 2001 to 74.8% in 2009, followed by a plateau in 2010 (76.3%) and 2011 (77.1%) as shown in Fig 5. A linear regression of the percentage of patients per year whose viral load was below detectable limit confirmed this growth. The slope of the regression line was 3.55 (t = 22.88 with a p-value of < 1 x 10^−8^. The regression line explained 97.8% of the variation in the data (adjusted R^2^). As with viral load, a linear regression demonstrated the significance of this growth in the mean CD4+ T cell counts in individuals in treatment over time. The slope of the regression line is 16.22 (t = 18.18 with a p-value of 3.77 x 10^−7^). This regression line explains 97.6% of the variation in the data.

Most likely in response to the increase in the rate of VLs below detection limit in individuals on treatment over time, there was an increase in the mean CD4+ T cell counts in individuals in treatment from 2001 to 2009, varying from 368 in 2001 to 414 in 2009 (S2 Table). By the end of 2011, 77.1% of individuals under ART in Brazil had VLs below 400 copies/mL of plasma. Among viremic individuals, 2.7% had VLs below 1,000 copies/mL, and 2.3% had VLs above 100,000 copies/mL. The percentages of VLs below 400 copies/mL were always lower among younger ages (t = -4.583 with 17.663 df and p = 1 x 10^−3^), but not statistically different between genders or among regions (Fig 5 and S1 Table).

**Fig 8:**
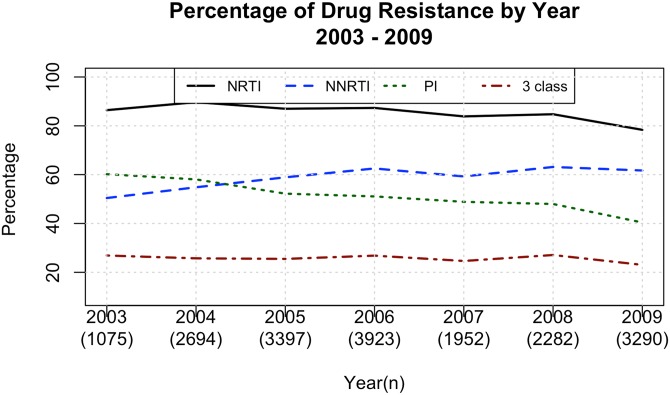
Antiretroviral resistance by class. Percentage of resistance to different antiretroviral classes and number of 3 class resistances among individuals in which the genotype test have been performed in Brazil from 2003 to 2009.

We next evaluated the impact of antiretroviral use and antiretroviral resistance over time at a population level. A previous analysis using the same data set with smaller sample size have already been published [4]. The percentage of NRTI resistance decreased over time (slope of regression line = -1.33, t = -2.937 with an R^2^ = 0.63 and p = 3.24 x 10^−2^) whereas resistance to NNRTIs increased (slope of regression line = 1.82, t = 3.621 with an R^2^ = 0.725 and p = 1.5x 10^−2^). PI resistance also decreased (slope of regression line = -2.96, t = -8.752 with an R^2^ = 0.939 and p = 3x 10^−3^) (Fig 8). The incidence of combined resistance to all three classes of antiretrovirals was stable over time at approximately 25%, that is, the value did not change significantly (t = -1.326 with an R^2^ = 0.260 and p = 2.42x 10^−1^). On average, 8% of the genomes sequenced were revealed to be wild type strains, which did not change over the time of analysis.

We were able to access the HIV-1 clade profile of 18,613 *pol* sequences for which genotype tests have been performed among individuals experiencing antiretroviral virologic failure. Overall, the most prevalent HIV-1 *pol* genotype was clade B, which accounted for 75.3% of sequences, followed by B/F recombinants (12.2%), C (5.8%), F (5.3%) and B/C recombinants (1.5%). We compared the percentage of patients in the South regions who exhibit clades B and C with the national proportions for the same clades to test if the South region represented a significantly different distribution of clades. The percentage for clade B appears significantly different than the concentration of this clade in all of Brazil. During the study period, clade B only represented 42.5% of patients in the south against 74.5% for the other regions. A two-sided t-test showed this to be significant with t = 6.43 on 7.9 df and a p-value of 2.2x 10^−4^ The C clade demonstrates an even greater difference among regions (34.5% of patients in the South versus 6.2% of patients in the other regions). This difference is significant as well (t = -5.91 on 6.7 df and a p-value of 6.9x 10^−3^).

**Fig 9:**
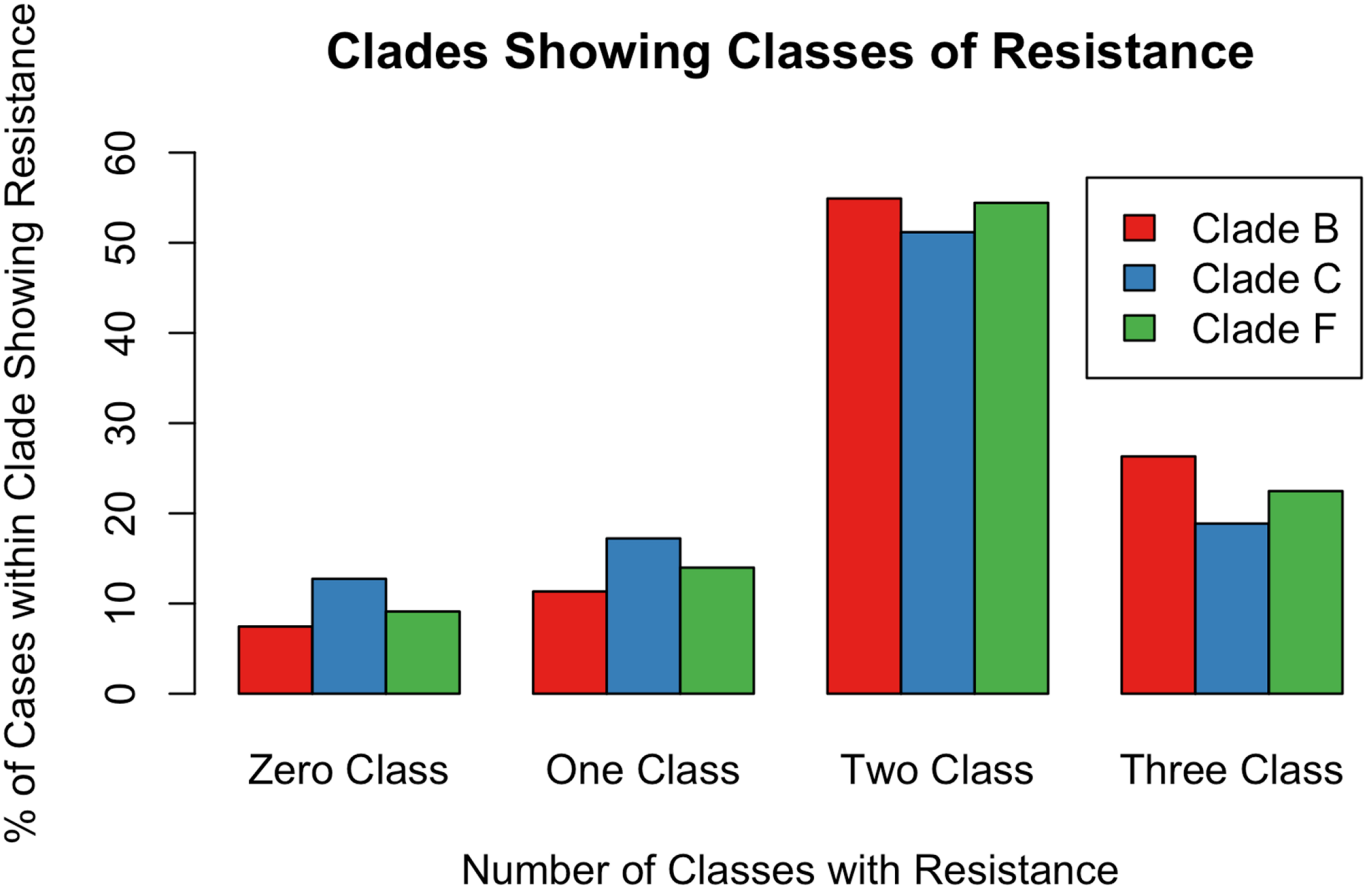
Antiretroviral class resistance by clade. Percentage of resistance to different antiretroviral classes by Clade (B, C and F) among patients treated between 2003 and 2009.

Combined resistance to three antiretroviral classes appears to be clade-related because it was higher on average among clade B (26.5%), followed by clade F (22.4%) and clade C (17.2%) viruses (t = 2.8005, with 7.3082 df and p = 2.54 x 10^−2^). Difference among clades for patients with resistance to two antiretroviral classes was not significant. Clade F was largest with 55.1% on average followed by 53.4% among clade B and 51.1% among clade C (t = -0.5968 with 7.7415 df and p = 0.5677. Resistance to one antiretroviral class was significant. The clade showing the highest rate of resistance to the antiretrovirals was Clade C with 16.1%. Clade F followed with 13.9% and clade B with 11.1% (t = 2.501, df = 11.673 and p = 2.830 x 10^−2^). There appears to be a difference among clades for patients who suffered no resistance to antivirals. Again, clade C predominated among these patients with 15.7%, followed by clade F (9.92%) and clade B (8.66%) (t = 2.2146 with 11.998 df and p = 4.69 x 10^−2^). Fig 9 shows these results.

## Discussion

Early detection of HIV cases is fundamental because initiating treatment to maximize survival and decrease HIV viremia in a population can also reduce HIV transmission [11]. In this study, more than one third of patients receiving their first HIV diagnosis already had critically low CD4+ T cell counts below 200 cells/mm^3^. This is higher than what was found in UK during the same period of time (23% between 1996 and 2010) [12], and similar to what has been described in New Zealand (32% from 2005–2010) [13], France (32.2% from 2003–2010) [14] and Portugal (35% from 2006–2014) [15]. Around 50% of individuals in our analysis presented CD4 levels below 350, which was also similar to what has been described in USA and Canada (between 38 to 46% from 1997–2007) [16] Ireland (54% between 2006–2008) [17], and Portugal (52% between 2006–2014) [15].

This analysis produced many interesting results. First, women presented with higher CD4+ T cell counts upon their initial HIV diagnosis, but this observation does not mean that this population looked for health assistance earlier than men, since uninfected women naturally tend to have higher CD4+ T cell counts than men [18]. In fact, the current study has not detected different rates of virological failure during ART between genders.

As also seen in USA and Canada [19], basal CD4 levels inversely correlated with age. Again, lower levels of CD4 cells among individuals older than 50 years of age may be related to natural age-related immunosenescence rather than delayed presentation for care. Alternatively, older people in this population might be infected for longer periods of time and their lower CD4 levels may reflect a longer-term disease progression. Although the lower basal CD4 levels in elderly people are particularly worrisome, response to ART has been more effective in this particular population in Brazil. HIV viral loads without treatment tends to be lower among women and elderly.

Our data also has some evidence that late presentation for care may be poverty-related. As described before, CD4 cell levels at presentation for care were higher in the Southeast region, followed by the South, Central West, Northeast and North regions. However, significant differences have been detected only between Southeast and Northeast regions. This exactly coincides with the level of social and economic development of each Brazilian region. However, we recognize that this is a limitation of the current study. We were not able to compare the immunological status of antiretroviral naïve individuals *vis-a-vis* HIV-1 subtype distribution since DNA sequencing analyses have been performed only among individuals experiencing antiretroviral virologic failure.

As expected, we detected an increase in ART virological efficacy over time. This reflects the improvements in antiretroviral drugs and treatment strategies over time. The immunological benefits at a population level can also be easily observed because the median CD4+ T cell counts in treated individuals increased over time. We note, however, that in 2001, less than 50% of individuals under antiretroviral therapy had VLs below 400 copies/mL (S1 Table). As in other countries, there was a trend in Brazil to replace treatment with unboosted PIs by boosted PIs, which tracks with the treatment efficacy trend. The use of boosted PIs also coincided with the increase of acquired antiretroviral resistance to PIs [20, 21]. Although local guidelines advocate that the initial ART should be NNRTI, the number of treatments using boosted PIs closely followed the number of treatments using NNRTI, reflecting the high number of patients on salvage therapy in this cohort.

As previously described, we also note that younger individuals need special attention since they had the poorest virological outcomes under ART [22]. This may relate to lower adherence among young adults due to more disordered lifestyles.

Although virological success rates have improved over time, nearly 23% of patients receiving ART were still viremic in 2011 (S1 Table). It can also be inferred that the majority of these viremic patients harbor antiretroviral-resistant strains because only 8% of patients on whom resistance testing was performed harbored wild type strains. 2.3% presented with VLs above 100,000 copies/mL. Although the antiretroviral resistance paradigm suggests that high VLs are usually related to wild type viruses and that resistance-related mutations generally have a fitness cost for HIV [23], as the number of resistance mutations exceeds a certain threshold, drug-resistant viruses can regain fitness. The high VL found in patients with drug-resistant strains demonstrates this [4]. This high VL in individuals harboring antiretroviral resistant viruses will theoretically increase the risk of transmitting drug resistance. Other studies have demonstrated that patients who harbor resistant HIV strains are more frequently involved in unsafe sex, thus exposing their partners to resistant HIV more frequently than individuals infected with wild type virus [24].

There was stable 3-class resistance over time. We should closely monitor this trend to create interventions to minimize the emergence of multi-drug resistant HIV strains. As our data shows, there is a differential prevalence of resistance to three antiretroviral classes among different clades, which may deserve further investigation.

Finally, the co-circulation of different HIV-1 clades in Brazil should be carefully followed up. The first HIV-1 recombinant strains were indeed described in Brazil [25]. Subsequently, a large number of HIV strains with recombinations between subtypes B, F and C have been described in Brazil. This has increased the genetic diversity of the epidemic in this particular country compared with North American and western European epidemics.

We recognize that the retrospective nature of our analysis and the absence of data such as the time of seroconversion for the analyzed individuals are limitations of this study. However, we were able to sort out a number of “real-life” features of the HIV epidemic in Brazil, a developing country with widespread access to antiretrovirals. These details reveal that we still need to improve our ability to diagnose HIV infection earlier and we need to focus more to the needs of women in treatment and the elderly population, as well as to less-developed regions. The efficacy of antiretrovirals, although improving, is still far from optimal, and antiretroviral resistance is a major problem.

## Supporting Information

S1 Table Characteristics of individuals by year at first presentation according to their CD4+ T cell counts and HIV-1 RNA VL. (XLSX)

S2 Table Percentage of VLs below 400 copies/mL over time in individuals under ART according to gender, Brazilian regions (Southeast, South, Central, Northeast, and North), and age. (XLSX)

## References

[1] Teixeira PR, Vitoria MA, Barcarolo J. Antiretroviral treatment in resource-poor settings: the Brazilian experience. AIDS. 2004;18 Suppl 3:S5–7. Epub 2004/08/24. 00002030-200406003-00002 [pii]. . 15322477 10.1097/00002030-200406003-00002

[2] Medeiros R, Diaz RS, Filho AC. Estimating the length of the first antiretroviral therapy regiment durability in Sao Paulo, Brazil. Braz J Infect Dis. 2002;6(6):298–304. Epub 2003/02/15. . 12585973 10.1590/s1413-86702002000600005

[3] Caseiro MM, Golega AA, Etzel A, Diaz RS. Characterization of virologic failure after an initially successful 48-week course of antiretroviral therapy in HIV/AIDS outpatients treated in Santos, Brazil. Braz J Infect Dis. 2008;12(3):162–6. Epub 2008/10/04. S1413-86702008000300001 [pii]. . 18833397 10.1590/s1413-86702008000300001

[4] Munerato P, Sucupira MC, Oliveros MP, Janini LM, de Souza DF, Pereira AA, et al HIV type 1 antiretroviral resistance mutations in subtypes B, C, and F in the City of Sao Paulo, Brazil. AIDS Res Hum Retroviruses. 2010;26(3):265–73. Epub 2010/03/10. 10.1089/aid.2009.0288 . 20210652

[5] Soares C, Vergara T, Sucupira MC, Brites C, Urbaez D, Pereira F, et al., editors. Prevalence of Transmitted HIV-1 Antiretroviral Resistance among Patients Initiating ART in Brazil: A Surveillance Using Dried Blood Spots. 18th Conference on Retrovirueses and Opportunistic Infections; 2011; Boston.

[6] Leal E, Silva WP, Sucupira MC, Janini LM, Diaz RS. Molecular and structural characterization of HIV-1 subtype B Brazilian isolates with GWGR tetramer at the tip of the V3-loop. Virology. 2008;381(2):222–9. Epub 2008/09/26. S0042-6822(08)00558-8 [pii] 10.1016/j.virol.2008.08.029 . 18814897

[7] Souza DC, Sucupira MC, Brindeiro RM, Fernandez JC, Sabino EC, Inocencio LA, et al The Brazilian network for HIV-1 genotyping external quality control assurance programme. J Int AIDS Soc. 2011;14:45 Epub 2011/09/23. 1758-2652-14-45 [pii] 10.1186/1758-2652-14-45 . 21936945 PMC3192700

[8] Wensing AM, Calvez V, Gunthard HF, Johnson VA, Paredes R, Pillay D, et al 2014 Update of the drug resistance mutations in HIV-1. Top Antivir Med. 2014;22(3):642–50. Epub 2014/08/08. . 25101529 PMC4392881

[9] Johnson VA, Brun-Vezinet F, Clotet B, Gunthard HF, Kuritzkes DR, Pillay D, et al Update of the drug resistance mutations in HIV-1: December 2010. Top HIV Med. 2010;18(5):156–63. Epub 2011/01/20. . 21245516

[10] R_Core_Team. R: A language and environment for statistical computing. In: R Foundation for Statistical Computing, editor. Vienna, Austria 2014.

[11] Granich RM, Gilks CF, Dye C, De Cock KM, Williams BG. Universal voluntary HIV testing with immediate antiretroviral therapy as a strategy for elimination of HIV transmission: a mathematical model. Lancet. 2009;373(9657):48–57. Epub 2008/11/29. S0140-6736(08)61697-9 [pii] 10.1016/S0140-6736(08)61697-9 . 19038438

[12] Iwuji CC, Churchill D, Gilleece Y, Weiss HA, Fisher M. Older HIV-infected individuals present late and have a higher mortality: Brighton, UK cohort study. BMC Public Health. 2013;13:397 Epub 2013/04/30. 1471-2458-13-397 [pii] 10.1186/1471-2458-13-397 . 23622568 PMC3651303

[13] Dickson N, McAllister S, Sharples K, Paul C. Late presentation of HIV infection among adults in New Zealand: 2005–2010. HIV Med. 2012;13(3):182–9. Epub 2011/11/19. 10.1111/j.1468-1293.2011.00959.x . 22093231

[14] Montlahuc C, Guiguet M, Abgrall S, Daneluzzi V, de Salvador F, Launay O, et al Impact of late presentation on the risk of death among HIV-infected people in France (2003–2009). J Acquir Immune Defic Syndr. 2013;64(2):197–203. Epub 2013/09/21. 10.1097/QAI.0b013e31829cfbfa 00126334-201310010-00013 [pii]. . 24047970

[15] Miranda AC, Moneti V, Brogueira P, Peres S, Baptista T, Aldir I, et al Evolution trends over three decades of HIV infection late diagnosis: the experience of a Portuguese cohort of 705 HIV-infected patients. J Int AIDS Soc. 2014;17(4 Suppl 3):19688 Epub 2014/11/15. 19688 [pii]. 10.7448/IAS.17.4.19688 25397438 PMC4225359

[16] Althoff KN, Gange SJ, Klein MB, Brooks JT, Hogg RS, Bosch RJ, et al Late presentation for human immunodeficiency virus care in the United States and Canada. Clin Infect Dis. 2010;50(11):1512–20. Epub 2010/04/27. 10.1086/652650 . 20415573 PMC2862849

[17] O'Shea D, Ebrahim M, Egli A, Redmond D, McConkey S. Late presentation of HIV despite earlier opportunities for detection, experience from an Irish tertiary referral institution. Ir J Med Sci. 2013;182(3):389–94. Epub 2013/01/17. 10.1007/s11845-012-0898-2 . 23322091

[18] Torres AJ, Angelo AL, Netto EM, Sampaio GP, Souza DF, Inocencio LA, et al Reference range for T lymphocytes populations in blood donors from two different regions in Brazil. Braz J Infect Dis. 2009;13(3):221–5. Epub 2010/03/02. S1413-86702009000300013 [pii]. . 20191201 10.1590/s1413-86702009000300013

[19] Althoff KN, Gebo KA, Gange SJ, Klein MB, Brooks JT, Hogg RS, et al CD4 count at presentation for HIV care in the United States and Canada: are those over 50 years more likely to have a delayed presentation? AIDS Res Ther. 2010;7:45 Epub 2010/12/17. 1742-6405-7-45 [pii] 10.1186/1742-6405-7-45 . 21159161 PMC3022663

[20] Daar ES, Tierney C, Fischl MA, Sax PE, Mollan K, Budhathoki C, et al Atazanavir plus ritonavir or efavirenz as part of a 3-drug regimen for initial treatment of HIV-1. Ann Intern Med. 2011;154(7):445–56. Epub 2011/02/16. 0003-4819-154-7-201104050-00316 [pii] 10.7326/0003-4819-154-7-201104050-00316 . 21320923 PMC3430716

[21] Riddler SA, Haubrich R, DiRienzo AG, Peeples L, Powderly WG, Klingman KL, et al Class-sparing regimens for initial treatment of HIV-1 infection. N Engl J Med. 2008;358(20):2095–106. Epub 2008/05/16. 358/20/2095 [pii] 10.1056/NEJMoa074609 . 18480202 PMC3885902

[22] Sabin CA, Smith CJ, d'Arminio Monforte A, Battegay M, Gabiano C, Galli L, et al Response to combination antiretroviral therapy: variation by age. AIDS. 2008;22(12):1463–73. Epub 2008/07/11. 10.1097/QAD.0b013e3282f88d02 00002030-200807310-00010 [pii]. . 18614870

[23] Deeks SG, Wrin T, Liegler T, Hoh R, Hayden M, Barbour JD, et al Virologic and immunologic consequences of discontinuing combination antiretroviral-drug therapy in HIV-infected patients with detectable viremia. N Engl J Med. 2001;344(7):472–80. Epub 2001/02/15. MJBA-440702 [pii] 10.1056/NEJM200102153440702 . 11172188

[24] Chin-Hong PV, Deeks SG, Liegler T, Hagos E, Krone MR, Grant RM, et al High-risk sexual behavior in adults with genotypically proven antiretroviral-resistant HIV infection. J Acquir Immune Defic Syndr. 2005;40(4):463–71. Epub 2005/11/11. 00126334-200512010-00013 [pii]. . 16280703 10.1097/01.qai.0000162238.93988.0c

[25] Sabino EC, Shpaer EG, Morgado MG, Korber BT, Diaz RS, Bongertz V, et al Identification of human immunodeficiency virus type 1 envelope genes recombinant between subtypes B and F in two epidemiologically linked individuals from Brazil. J Virol. 1994;68(10):6340–6. Epub 1994/10/01. . 8083973 10.1128/jvi.68.10.6340-6346.1994 PMC237055

